# Gut associated metabolites and their roles in *Clostridioides difficile* pathogenesis

**DOI:** 10.1080/19490976.2022.2094672

**Published:** 2022-07-04

**Authors:** Andrea Martinez Aguirre, Joseph A. Sorg

**Affiliations:** Department of Biology, Texas A&M University, College Station, TX, USA

**Keywords:** *Clostridioides difficile*, gut metabolites, microbiome, fecal microbial therapy, bile acids, Stickland, short-chain fatty acids

## Abstract

The nosocomial pathogen *Clostridioides difficile* is a burden to the healthcare system. Gut microbiome disruption, most commonly by broad-spectrum antibiotic treatment, is well established to generate a state that is susceptible to CDI. A variety of metabolites produced by the host and/or gut microbiota have been shown to interact with *C. difficile*. Certain bile acids promote/inhibit germination while other cholesterol-derived compounds and amino acids used in the Stickland metabolic pathway affect growth and CDI colonization. Short chain fatty acids maintain intestinal barrier integrity and a myriad of other metabolic compounds are used as nutritional sources or used by *C. difficile* to inhibit or outcompete other bacteria in the gut. As the move toward non-antibiotic CDI treatment takes place, a deeper understanding of interactions between *C. difficile* and the host’s gut microbiome and metabolites becomes more relevant.

## Introduction

*Clostridioides difficile* is a Gram-positive, pathogenic, spore forming, anaerobic bacterium that is considered the main cause of antibiotic-associated diarrhea, pseudomembranous colitis and toxic megacolon.^1^ According to the Centers for Disease Control and Prevention (CDC), *C. difficile* is a major nosocomial pathogen with more than 220,000 infections, 13,000 deaths, and nearly $5 billion in annual treatment associated costs that are predicted to increase in the future.^2,3^ This, combined with its inherent natural antibiotic resistance, led to the CDC classifying *C. difficile* as an ‘urgent threat’ to the United States healthcare system. The greatest risk factor for *C. difficile* infection (CDI) is prior treatment with broad-spectrum antibiotics. Antibiotics render the host susceptible to CDI by changing the ecology of the microbiota, which is known to provide ‘colonization resistance’ against invading pathogens, *C. difficile-*included.^4,5^ Interestingly, infants and newborns can be *C. difficile* carriers^6^ and *C. difficile* can be found in approximately 53% of healthy adults.^7^ Thus, *C. difficile* could be considered a part of the microbiome in humans, but this is mostly defined by age.^8^

Endospores are metabolically dormant forms of spore-forming bacteria produced in response to stress (*e.g*., nutrient limitation).^9–11^ For *C. difficile*, the spore form is essential for host-to-host transmission due to the strictly anaerobic nature of the vegetative form.^12–14^ Thus, in susceptible hosts, *C. difficile* spores must germinate into the active, vegetative form in order to multiply and cause disease. Germination by *C. difficile* spores is triggered upon recognition of certain bile acids and amino acids by germinant receptors.^11,15,16^

Dormant *C. difficile* spores are considered infectious agents, but the growing *C. difficile* vegetative cells secrete the TcdA and TcdB toxins that are responsible for the primary symptoms of disease (*e.g*., diarrhea and colitis);^14,17–19^ while some *C. difficile* strains secrete a third toxin, a binary toxin.^20^ The most common treatments for CDI are broad-spectrum antibiotics like vancomycin, although fidaxomicin (a narrower-spectrum antibiotic)is now recommended for initial and recurrent CDI . Unfortunately, the higher cost of fidaxomicin is limiting its use, and the continued alteration to the colonic microbiota by these antibiotics, lead to patients experiencing recurring disease due to the presence of *C. difficile* spores within the gastrointestinal tract or in the surrounding environment.^21,22^

Recently, fecal microbial transplantation (FMT) has emerged as an effective treatment against *C. difficile*, especially for patients with recurrent CDI. FMT is hypothesized to drive protection against CDI through the restoration of gut microbes, gut associated metabolites [*e.g*., secondary bile acids and short chain fatty acids (SCFA)]^23^ and/or microbial bile acid biotransformation . Nevertheless, the use of FMT is not without risk for the potential introduction of harmful microbes to the recipient.^24–26^ Thus, effective and safe treatments for CDI that result in the restoration of the protective metabolic effects provided by the microbiome still need to be developed. In this review, we summarize studies focusing on metabolites produced by the host or gut microbiota and how these molecules influence *C. difficile* pathogenesis.

## Bile acid metabolites and their role in *C. difficile* pathogenesis

**Figure 1.**
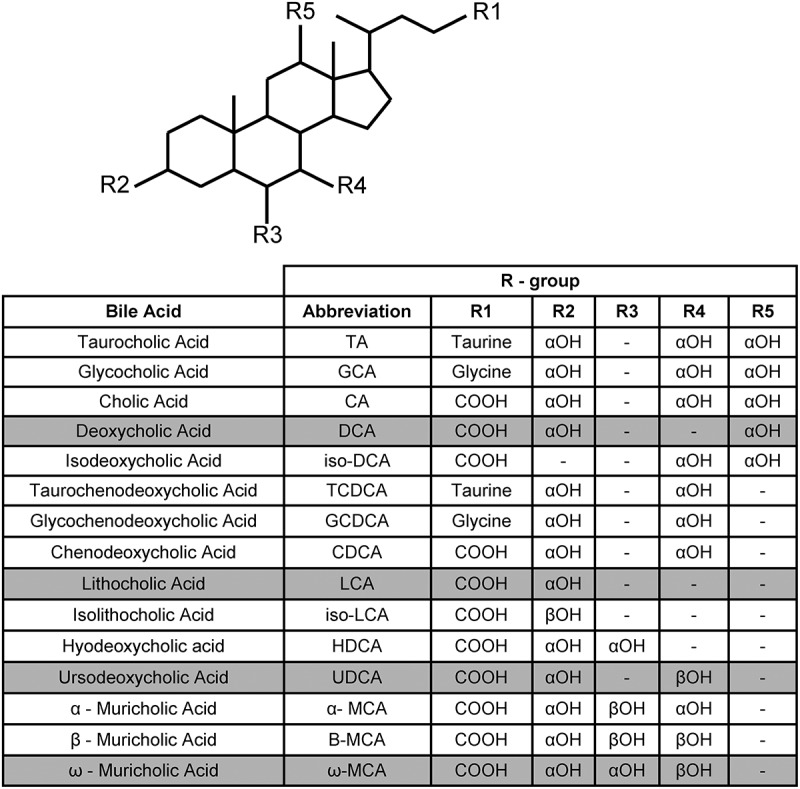
Structures of the bile acids discussed in this review.

Bile acids are cholesterol-based molecules synthesized by hepatocytes in the liver that play key roles in regulating metabolic pathways and aiding in the absorption of fats and cholesterol during digestion.^27,28^ There are two pathways for bile acid synthesis: the classical pathway generates both cholic acid (CA) and chenodeoxycholic acid (CDCA) while the alternative bile acid synthesis pathway only forms CDCA.^29^ Before being secreted into the intestines, primary bile acids are conjugated with either taurine or glycine at C-24 to generate taurocholic acid (TA)/taurochenodeoxycholic acid (TCDCA) and glycocholic (GCA) acid/glycochenodeoxycholic acid (GCDCA) (Figure 1).^30,31^ Reabsorption of bile acids takes place throughout the intestines and the reabsorbed bile acids are recycled back to the liver for additional rounds of digestion.^29,31^ During this enterohepatic recirculation pathway, bile acid synthesis is regulated through the Farsenoid X receptor (FXR) transcriptional activator^32^ . Bile acid binding principally through CDCA to FXR generates a conformational change in FXR that leads to the synthesis of FGF19 in illeal enterocytes^32,33^ . FGF19 is secreted by the ileal enterocytes and binds to the FGFR4 receptor on the cell surface of hepatocytes. Binding of FGF19 to the receptor leads to a negative feedback regulation on bile acid synthesis.^32,33^ In an FMT model for recurrent CDI patients, levels of FGF19 were significantly increased post-FMT, thus suggesting upregulation of the FXR-FGF pathway after FMT that may aid in *C. difficile* clearance.^34^ Furthermore administration of obeticholic acid, an FXR agonist, to HFD CDI-infected mice resulted in decreases in disease severity, and the use of ursodeoxycholate (UCA) in a CDI mouse model showed increased levels of FXR as well as TGR5 that modulates the immune response against *C. difficile*.^35,36^ The mentioned studies on FXR suggest that FXR modulation may be important for CDI treatment but further research is necessary to move past the correlation observed in FXR that shows protection against CDI.

Shaded rows indicate secondary bile acids. Unshaded rows indicate conjugated and deconjugated primary bile acids.

A small percentahe of bile acids escape enterohepatic recirculation, and can be deconjugated by the bile salt hydrolases that are expressed in many gut microbes. Subsequently, deconjugated bile acids can be 7α-dehydroxylated by a small subset of gut microbial species to generate secondary bile acids (deoxycholic acid (DCA), from CA, and lithocholic acid (LCA) from CDCA) (Figure 1).

Cholic acid-derived bile acids promote *C. difficile* germination, whereas bile acids of the CDCA family act as inhibitors of C. *difficile* germination.^16,37,38^ In addition to acting as activators/inhibitors of *C. difficile* spore germination, bile acids affect *C. difficile* vegetative growth. The host-derived, primary, bile acids TA and CDCA have different effects on *C. difficile* growth. Vegetative cell growth seems to be unaffected by TA, however *C. difficile* growth is strongly inhibited by CDCA.^16^ Interestingly, DCA, a product of the 7α-dehydroxylation of CA by the colonic microbiota, is also toxic to *C. difficile* growth in-vitro, suggesting that the 7α-dehydroxylation of CA by the colonic microbiota may prevent *C. difficile* from colonizing healthy hosts.^16^ When the minimal inhibitory concentrations (MIC) of various bile acids were determined, the MIC of CA was 10 mM for 2 different *C. difficile* strains, a concentration that is not found in the GI tract, thus not physiologically relevant.^39^ However, increasing the hydrophobicity of the base CA structure (which has three hydroxyl groups, Figure 1), by removing either the 12α hydroxyl (generating CDCA) or the 7α hydroxyl (generating DCA) resulted in the MIC decreasing from 10-fold to 1 mM.^39^ Moreover, the 7β-epimer of CDCA, UCA) (Figure 1), inhibits growth of *C. difficile* cells,^40^ although over a 24-h time period, *C. difficile* grew in the presence of 2 mM UCA.^41^ Because CDCA completely inhibited the growth of *C. difficile* vegetative cells over a 24-h period and UCA did not, this could imply that stereochemistry of the bile acid structure plays a role in toxicity.^39,40^ Importantly, though these two studies were done using different *C. difficile* isolates, which may have different MIC values for bile acids as observed for other *C. difficile* strains.^39^

Mice are one of the most common animal models used in CDI studies and their murine bile acid composition and their effects on CDI are well understood.^39,42^ In rodents, the presence of alternative hydroxylating enzymes yields other primary bile acids (α/β/ω-muricholic acids) (Figure 1).^43^ Muricholic acids have a MIC similar to that of CDCA and DCA.^39^ Similar to CA, muricholic acids are also trihydroxyl bile acids but the hydroxyls are in the 3, 6, and 7 positions instead of the 3, 7, and 12 positions that are found on CA-derived bile acids (Figure 1).^39^ A study found that in addition to the bile acids described above, isodeoxycholic acid (a 7α, 12α-hydroxy bile acid), lithocholic acid (a 3α-hydroxy bile acid), isolithocholic acid (a 3β-hydroxy bile acid), and hyodeoxycholic acid also inhibit the growth of *C. difficile* vegetative cells.^44^ Furthermore, isoallolithocholic acid (isoalloLCA), the isomer of isoLCA had an MIC_90_ of 2 µM in *C. difficile* CD630 (a laboratory strain) and in the highly toxigenic *C. difficile* VPI10463 strain.^45^

Due to the inherent toxicity of secondary bile acids for *C. difficile* growth, it was hypothesized that their production would toxify an environment and limit *C. difficile* growth.^16,46^ To test this hypothesis, Theriot and Young^5^ showed evidence that the presence of secondary bile acids is responsible for protection against CDI. Using a multi-omics approach, the authors identified *C. difficile* resistant and susceptible states in an antibiotic-treated murine model. A susceptible state correlated with high levels of conjugated primary bile acids (*e.g*., TA) whereas a resistance state correlated with higher levels of secondary bile acids (*e.g*., DCA). Later, Buffie et al.^4^ also provided compelling data in support of this hypothesis. Using mouse models and human subjects, the authors found that high levels of DCA or the presence of the genetic operon that is known to generate secondary bile acids (*bai*), strongly correlated with a protective environment. Additionally, mice treated with different antibiotics resulted in variable metabolic profiles and certain secondary bile acids (*i.e*., omega-muricholic acid, LCA and, to a lesser extent, hyodeoxycholic acid, and UCA) that could inhibit TA- and DCA-mediated colony formation by *C. difficile* spores.^16,38,39,47^

These landmark studies demonstrated that increased secondary bile acids and the genes encoding proteins responsible for their production strongly correlate with disease-resistant states and increased primary bile acids correlate with disease-susceptible states. A more recent article^45^ analyzing metabolites in centennials collected stool from centennials(~107 year olds), elderly adults (85–89 years old), and young adults (21–55 years old) and bile acids were measured. The authors observed higher levels of isoLCA, 3-oxoLCA, alloLCA, 3-oxoalloLCA, and isoalloLCA in the centennial samples, when compared to the other study population. Additionally, the authors characterized the biosynthetic pathways to generate these CDCA derivatives and suggested that further elucidation of microbial biotransformations may expand our understanding of intestinal homeostasis and, as a result, possible protection effects of additional bile acids against CDI^45^

Interestingly, a recent study using a cholate-deficient mouse strain showed that the cholate-derived secondary bile acid (DCA) is dispensable for protection against CDI, and that lack of the bile acid transcriptional activator FXR, does not show differences in CDI mice. Also, when germ free mice were monoassociated with bacteria known to generate secondary bile acids, no secondary bile acids were present in fecal samples, but the mice were still protected against CDI.^48^ This emerging evidence would suggest that although bile acids are important for *C. difficile* colonization to be established in the gut, these metabolites may not play such an important role in generating a protective environment against *C. difficile*. In support of this, a 2018 study^49^ showed that the levels of the conjugated primary bile acids TA and TCDCA decreased 2 days post-FMT. In contrast, there was an increase of the secondary bile acids DCA, LCA, and UDCA.^49^ Interestingly, the authors note that although levels of secondary bile acids increased, the presence of secondary bile acids was not indicative of FMT success or failure, thus suggesting that although bile acids may play a role in protection, there are other mechanisms driving CDI resistance.^49,50^

Regardless, *C. difficile* encounters bile acid during colonization and its growth in the gut may impact bile acid synthesis in the liver. Taking advantage of MALDI imaging mass spectrometry, Wexler et al.^51^ found the levels of primary bile acids in the mouse gut increased significantly as early as one-day post-CDI. Additionally, the authors found that the introduction of cholestyramine, a bile-acid sequestering drug, lead to delayed *C. difficile* colonization.^51^ Their results suggest that primary bile acids are required to efficiently establish *C. difficile* in a host.

Regarding secondary bile acids, in addition to its toxic effect against *C. difficile* vegetative cells, the secondary bile salt DCA induces *C. difficile* biofilm formation at low concentrations (240 µM) in a non-biofilm-producing *C. difficile* CD630∆erm strain.^52^ Thus, it is possible that a decrease in bile acid levels during late infection stages may generate favorable conditions for biofilm formation. Indeed, the authors hypothesized that the low concentrations of the bile salt encountered during the natural restoration of microbiome may allow the cells to transition toward biofilm formation. This would protect *C. difficile* vegetative cells against concentrations of antibacterial compounds that may normally inhibit growth (*i.e*., antibiotics or other bile acids).^52^

In addition to promoting germination and inducing biofilm formation, bile acids can alter *C. difficile* physiology in other ways. Low amounts of LCA (0.08 mM) resulted in more elongated vegetative cells and absence of flagella. Fewer flagella were also observed when vegetative cells were incubated in 0.8 mM DCA and 0.3 mM CDCA. The authors hypothesized that lack of flagella may allow for better adherence to intestinal lining during infection. Additionally, bile acids resulted in up-regulation of the chaperon proteins DnaK, DnaJ, GrpE, GroL, and GroS, but the mechanism of up-regulation for chaperones is still unknown.^53^

Finally, bile acids inhibit TcdB toxin activity by binding to the toxin. Both conjugated and deconjugated secondary bile acids (e.g., DCA, LCA, GDCA, TDCA, GLCA, and TLCA) have greater potency in inhibiting toxin activity than their primary bile acid counterparts, but toxin binding by bile acids is reversible. The reversible binding and inhibition of bile acids to *C. difficile* toxins may thus suggest modulation mechanisms taking place in the gut depending on bile acid composition in the host.^54^

The role of bile acids in relation to *C. difficile* physiology and pathogenesis has been studied and characterized, extensively, since the discovery of their function as germinants for *C. difficile* spores.^16,46^ Because of the many functions bile acids play in the host, it is not surprising that the interaction with *C. difficile* is complex and multifaceted, with both positive and negative interactions observed – depending on the conformation of, the abundance of, and the location of specific bile acids as well as the *C. difficile* life cycle stage in the host. More work should be done that move the field from data that correlate what bile acids are present in the host to potential causative interactions between bile acids and the host/*C. difficile*.

## Stickland metabolites, amino acids, and the Wood Ljundahl pathway in *C. difficile* pathogenesis

**Figure 2.**
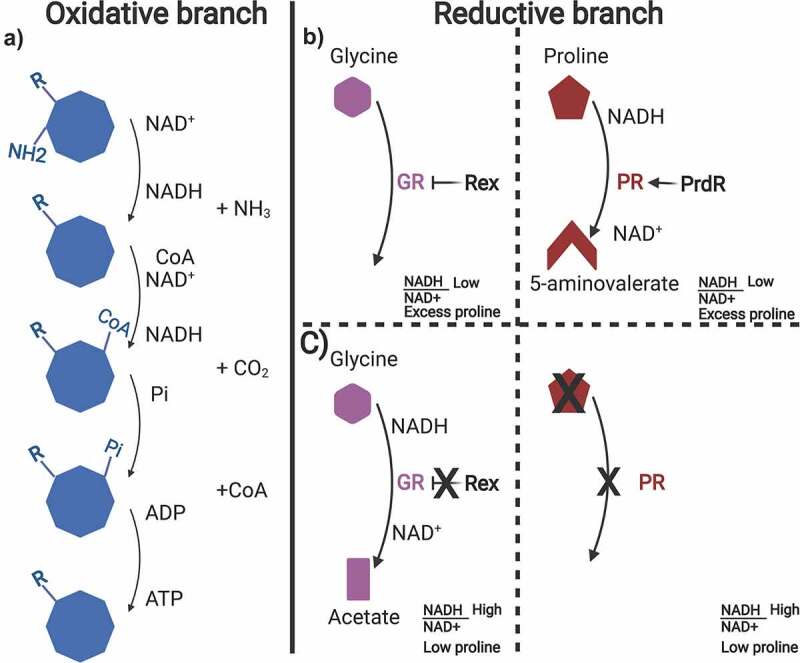
Oxidative and reductive branch of Stickland metabolism in *C. difficile.*

Stickland metabolism was first identified in 1934 as the predominant pathway for *Clostridium sporogenes* energy production.^55^ Stickland metabolism couples pairs of amino acids that may act as electron donors or acceptors.^56,57^ The donor amino acid is oxidatively deaminated or decarboxylated to produce NADH, whereas the electron accepting amino acid is reduced to regenerate NAD^+^ (Figure 2(a)). Although a myriad of amino acids can be used in the oxidative branch, the reductive branch is fueled only by proline or glycine. In the reductive branch, proline is consumed by proline reductase (PrdB, PrdA), a selenium-containing enzyme (selenoenzyme) that generates 5-aminovalerate and NAD^+^, and glycine is consumed by glycine reductase (GrdA, GrdB), selenoproteins that generate acetate and NAD^+^ (Figure 2(b,c)).^58,59^

Graphical representation of the oxidative and reductive Stickland pathways. A) Oxidation of a myriad of amino acids takes place in the oxidative branch of Stickland metabolism resulting in 2 NADH molecules and 1 ATP molecule produced. B) Proline and glycine are used to regenerate NAD^+^ in the reductive branch. When excess proline is available in the surrounding environment and the NADH/NAD^+^ ratio in the cell is low, the PrdR activator promotes proline reductase (PR) expression to generate 5-aminovalerate and NAD^+^. Concurrently, Rex inhibits glycine reductase (GR). C) When proline levels are low and the NADH/NAD^+^ ratio is high, Rex is unable to inhibit glycine reductase allowing acetate formation. Proline reductase activity is not present. Created with BioRender.com.

Although Stickland metabolism is well-characterized in *Clostridium sticklandii*,^60^ this alternative metabolic pathway in other Clostridial species has just recently been elucidated. Bouillaut et al.^59^ analyzed the *C. difficile* glycine and proline reduction operons and their activity in the reductive pathway of Stickland metabolism. A mutation in the *prdB* subunit of the proline reductase enzyme resulted in a decrease in growth in rich media, but a mutation in a *grdA* mutant did not affect growth in rich medium.^59^ Additionally, the authors characterize the sigma-54 dependent activator, PrdR that acts as a mediator for PrdB-dependent activation and proline-dependent toxin repression.^59^ In addition, given the role the Stickland reductive branch has in regenerating NAD^+^ for the cell, the redox dependent transcriptional repressor, Rex, has a role in proline-dependent regulation and is controlled by PrdR in *C. difficile*.^61^ Using DNA binding assays and qRT-PCR, the authors found that proline seems to be the preferred amino acid for regeneration of NAD^+^. In presence of excess proline, PrdR stimulates proline reductase expression and simultaneously Rex represses the glycine reductase gene *grdE*. NADH is then oxidized and, as a result, the ratio of NADH/NAD^+^ is low. Alternatively, when levels of proline are low, the NADH/NAD^+^ ratio increases and the high levels of NADH prevents Rex from repressing glycine reductase expression (Figure 2(b,c)).^61^

Selenium is an essential component of the proline and glycine reductases. Selenium is incorporated into these proteins as selenocysteine. Selenocysteine is generated through a synthesis pathway where the product of the *selD* gene reacts inorganic phosphate with hydrogen selenide to generate selenophosphate.^58,62,63^ A *selD* mutant is unable to incorporate selenium into proteins and this results in a complete loss of the reductive branch of Stickland metabolism. The loss of selenophosphate generation results in a defect in the ability of *C. difficile* spores to outgrow following germination in peptide-rich media.^63,64^ Also, the absence of selenophosphate altered *C. difficile* physiology so that other NAD^+^ regeneration pathways were expressed.^64^

Several studies have correlated the depletion or increased levels of amino acids important for Stickland metabolism using both *in vitro* and *in vivo* approaches.^48,65–68^ In an *in vitro*, multi-omics approach, the metabolome of multiple *C. difficile* growth stages found that Stickland metabolites were dramatically depleted upon entry into stationary phase^69^ . Furthermore, in stool samples derived from hospitalized patients, amino acids used for Stickland metabolism were depleted in CDI patients, suggesting that these amino acids were consumed by *C. difficile* vegetative cells. In this study, branched chain amino acids, such as leucine, were hypothesized to be the preferred amino acid used by *C. difficile*; other amino acids, such as proline, tyrosine, and phenylalanine are possible sources of energy for both *C. difficile* and other gut microbes.^67^

Proline availability is important for C. *difficile* colonization in mice. A mutation in proline reductase (*prdB*) resulted in decreased colonization in a humanized microbiome mouse model.^65^ Moreover, *C. difficile* can take advantage of inflammation-induced collagen degradation. During this toxin-dependent process, the host cells respond to this inflammation by producing matrix metalloproteases. This results in the degradation of collagen – a protein rich in proline.^70^

Building upon this, in mice monoassociated with *C. scindens, C. hiranonis*, or *C. leptum*, proline was greatly depleted and mice were protected against CDI.^48^ This work is also supported by *in vitro* conditions where *C. difficile* and *C. hiranonis* compete for nutrients.^71^ Interestingly, Battagliogli et al.^65^ observed high levels of the amino acids glycine, proline, threonine, and alanine in a dysbiotic colon, whereas Aguirre et. al.^48^ observed depletion of proline and glycine in their monoassociated mice (amino acids essential for the reductive pathway of Stickland metabolism).^48,65^ Nevertheless, direct comparison should not be made since one study used a humanized microbiome mouse model and the other used germ-free mice that were monoassociated with individual bacteria.

Expanding on the hypothesis that depletion of amino acids is important for Stickland metabolism, and may drive protection against CDI, Girinathan et al.^66^ found that the gut commensal bacterium *P. bifermentans* protects against CDI, at least in part, through depleting nutrient sources used by *C. difficile*. Using *P. bifermentans* monoassociated mice, as well as carbon-source enrichment analysis of the gut-metabolomic environment, the authors found alterations in the substrates/products of Stickland metabolism (such as proline, glycine, threonine and 4-hydroxyproline) and production of 5-aminovalerate.^66^

Although *in vivo* FMT studies have reported levels of amino acids in FMT-recipients, such as increasing levels of valine, isoleucine, and leucine (amino acids able to be used in the oxidative branch), the presence of Stickland products in these recipients have not been reported [besides the SCFA (acetate, propionate)].^72–74^ In a chemostat model, levels of 5-aminovalerate decreased post-FMT and isobutyrate levels remain constant post-FMT. A plausible explanation for the observed 5-aminovalerate levels could be the lack of Stickland fermenters included in the stable communities introduced into the chemostat, such as *P. bifermentans*.^75^

Expressed at lower levels when other forms of energy production are present, the Wood-Ljungdahl Pathway (WLP) may need examining during CDI. Through the WLP, two molecules of CO_2_ are reduced to acetate.^76^ The WLP can be coupled with butyrate production to allow for increased efficiency of ATP formation by decreasing nutritional requirements. Thus, the WLP may prove useful when glucose or amino acid levels are low, and *C. difficile* may need to adapt to low nutrient conditions.^77^ Moreover, in a mouse model of CDI, the WLP increased expression twenty-four hours post-infection.^78^ This suggests more attention should be given to this pathway in animal models, or in patient cohort samples.

Stickland metabolism by *C. difficile* during host colonization is well-established.^48,65,68^ Nevertheless, the availability of the substrates needed for the oxidative and reductive branches of Stickland metabolism, and required compounds for WLP, during CDI likely shapes the ability of the bacterium to cause disease. Similarly, the presence of Stickland-using microbial competitors in the host microbiome also shapes how *C. difficile* colonizes a host.^48,66,79^ Therefore, a better understanding of specific conditions in which *C. difficile* takes advantage of alternative metabolic pathways is necessary for the study of novel therapeutics that may modulate these pathways.

## Non bile acid metabolites that correlate or show protection against *C. difficile* pathogenesis

**Figure 3.**
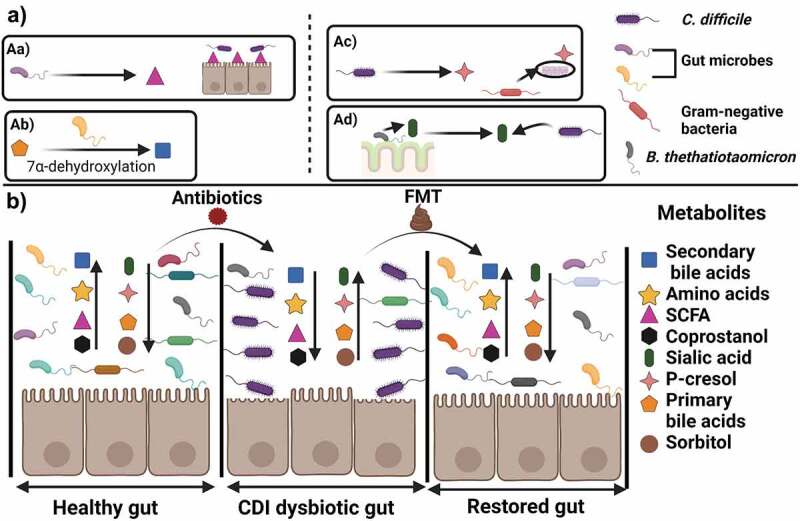
Gut metabolites effect in *C. difficile* growth and colonization.

The gut microbiome is complex, with interactions occurring between resident and invading bacteria that result in the generation of secondary metabolites. One such metabolite is coprostanol. Coprostanol is generated through the reduction of the double bond between C5 and C6 of cholesterol.^80^ In the metabolome of healthy controls vs. CDI patients, 63 bacterial OTUs were identified that positively correlated with the presence of coprostanol, which in turn negatively correlated with CDI patients.^81^ The majority of phylotypes that correlated with coprostanol presence were members of the Lachnospiraceae and Ruminococcaceae families. Coprostanol may enhance resistance to CDI by decreasing the availability of cholesterol which could reduce the abundance of metabolites (*i.e*., primary bile acids) that are necessary for germination by *C. difficile* spores (Figure 3(a,b).^81^

A) Examples of mechanisms of inhibition or protection against CDI. Aa) Gut microbes produce SCFA that aid in intestinal barrier function. Ab) Bacteria that perform 7α-dehydroxylation generate secondary bile acids that correlate with CDI resistant states. On the other hand Ac) generation of p-cresol by *C. difficile* may affect barrier function in Gram-negative bacteria, Ad) release of sialic acid from the colon mucus layer is used as a nutrient source by *C. difficile*. B) In an undisrupted gut environment amino acid, SCFA and secondary bile acid levels are high, creating a resistant state against *C. difficile* through immune defenses and nutrient limitation. High levels of the cholesterol derivative, coprostanol, are observed indicative of limited availability of primary bile acids. Introduction of antibiotics compromises the gut environment, and *C. difficile* toxins disrupt the intestinal barrier. *C. difficile* also promotes increasing levels of p-cresol, conjugated primary bile acids (*e.g*. TA) and sorbitol as well as use sialic acid as a nutrient source. FMT treatment allows restauration of gut metabolites and restore a resistant state against CDI. Created with BioRender.com.

Three microbial-derived SCFAs (propionate, acetate, and butyrate) have long been associated with CDI resistance.^82–85^ In general, SCFAs are believed to play important roles in maintaining gut homeostasis.^86^ Several studies have shown that SCFAs are depleted during CDI^5,81,86^ Recently, butyrate, and acetate were found to protect against CDI by aiding in maintaining intestinal barrier integrity.^87,88^ Butyrate plays a role in maintaining intestinal barrier integrity through limiting the permeability of intestinal epithelial cells to *C. difficile* toxins by stabilizing the hypoxia-inducible factor 1 alpha (HIF-1α).^87^ Acetate acts during the early stages of *C. difficile* infection by activating the free fatty acid receptor 2 (FFAR2) signaling pathway by augmentation of IL-1B production from neutrophils and IL-1 R through signaling of ILC3. These two types of immune cells then induce production of IL-22 that is implicated in antimicrobial and repair mechanisms in intestinal epithelial cells.^88^

The mechanism by which propionate confers protection against CDI has yet to be elucidated. However, taking from data published on butyrate and acetate, a hypothesis could be that propionate acts upon specific host immune factors (Figure 3a). Interestingly, Gregory et al.^89^ suggested that increasing levels of SFCAs as the gut microbiome recovers from disruption, may be used by *C. difficile* vegetative cells as a triggering signal to upregulate toxin secretion and promote the inflammation that would allow the bacterium to maintain colonization in the gut.^89^

A recent study that used^1^H-NMR on stool samples derived from recurring CDI patients that received FMT, through capsule or colonoscopy, found increased levels of acetate, butyrate, and propionate in recipients 12-weeks post-FMT.^85^ Interestingly, valerate, another SCFA was depleted in a chemostat C. *difficile* infection model.^75^ Similar to the other SCFA, valerate levels were restored upon FMT. The study also found that valerate inhibited *in vitro C. difficile* vegetative growth in a dose-dependent manner, and that introducing valerate orally in the form of 15 mM glycerol trivalerate into a *C. difficile* mouse model decreased total viable counts.^75,85^ It is important to note, though, that the glycerol trivalerate given to the mice was above physiologically relevant concentration since the authors found that chemostat FMT cultures only reached 4 mM valerate.

In addition to SCFAs, the microbiome produces antimicrobial compounds. Two Clostridial species, *C. scindens* and *C. sordellii* secrete tryptophan-derived antibiotic compounds (*i.e*., 1-acetyl-β-carboline and turbomycin A) that inhibit *C. difficile* growth.^90^ Interestingly, the anti-*C. difficile* effect of the antibiotics was enhanced by DCA and LCA. These findings suggest that a combination of secondary bile acids as well as antibiotics is at play during prevention of disease.^90^ However, a recent study with mice that were monoassociated with *C. scindens* did not detect 1-acetyl-β-carboline in their metabolomics analysis,^48^ suggesting that the activity of 1-acetyl-β-carboline may require specific conditions to be produced.

Dynamic interactions between gut microbes are constantly taking place and involve the production of and the sensing of secondary metabolites.^91^ Although the mechanisms by which some of these metabolites protect against CDI are still unclear,^81,87,88,90^ their identification provides important data that can be directly tested as potential treatment options for CDI. Moreover, a microbiome-centric approach for CDI treatment will most likely find other metabolites that show protection against CDI and could be explored for therapeutic approaches.

## Metabolites and their beneficial roles for *C. difficile* infection

In recent years, the research focus on the modulation of the microbiome by *C. difficile* and how the bacterium survives in the host has increased.^92–96^ *C. difficile* can produce products that promote inflammation and that have antibacterial effects. *C. difficile* generates *p*-cresol, a phenolic compound that affects the integrity of surface barriers in bacterial cells with a higher effect observed in Gram-negative bacteria.^93^ The *hpdBCA* operon is responsible for the fermentation of tyrosine to *p*-hydroxyphenylacetate to then generate *p*-cresol using the 4-hydroxyphenylacetate dehydrogenase enzyme. In a mouse model of recurrent CDI, mice infected with a *hpdC* mutant strain, had increased microbial diversity compared to mice infected with the wildtype strain, as well as lower *C. difficile* viable counts. Additionally, when exogenous *p*-cresol was introduced to healthy human fecal slurries, the number of viable total anaerobes increased, thus suggesting that generation of *p*-cresol by *C. difficile* may give the bacterium a competitive advantage over other gut microbial species (Figure 3(a,c)).^93^

Similar to the production of *p*-cresol, production of sorbitol by *C. difficile* may enhance colonization. A *C. difficile* strain that is unable to produce sorbitol is outcompeted 10-fold by its wildtype counterpart in a mouse model.^94^ Upon further investigation, the authors found that the inflammation induced by the *C. difficile* toxins results in an upregulation of aldose reductase which generates host-derived sorbitol. These results show how *C. difficile* is able to use a diet and host-derived nutrient to expand in the perturbed microbiome environment (Figure 3(b)).^94^

*C. difficile* also can use metabolites produced by the host and other gut microbes during colonization.^92,95,96^ The gut symbiont *Bacteroides thetatiotaomicron* encodes a sialidase enzyme that cleaves and releases sialic acid from mucosal glycoconjugates. *C. difficile* encodes a sialic acid catabolic operon. Using a transcriptional analysis of germ free and *B. thetatiotaomicron* monoassociated mice, the data showed that the ability of *B. thetatiotaomicron* to release sialic acid resulted in increased expression levels of the *C. difficile* sialic acid operon.^95^ Additionally, a spike in free sialic acid 1 day after antibiotic treatment of mice was observed but these levels reduced 3 days after antibiotic treatment.^95^ Moreover, mice monoassociated with a *B. thetatiotaomicron* sialidase mutant strain and then infected with *C. difficile* had lower *C. difficile* CFU counts (Figure 3(a-d)).^95^ These results suggest an important role of sialic acid during *in vivo* infection.

Another host-produced metabolite, heme, can be used by *C. difficile*.^92^ The heme-sensing membrane protein system, HsmRA, protects against redox damage generated from antibiotic treatment of CDI. *C. difficile* HsmA binds to heme that is released from the inflamed GI tract and shields the bacterium from redox-active molecules. Although the specific mechanism of protection by HsmA is still unknown, the authors hypothesized the use of HsmRA by other pathogens for protection against oxidative stress may be taking place.^92^

Finally, indole may play a role during *C. difficile* infections. Supernatant from *C. difficile* stationary phase cultures can induce the expression of tryptophanase (*tnaA*) in *E. coli*. The levels of indole increased in other indole-producing microbes in the gut (*e.g., L. reuteri* and *E. faecalis*) in co-culturing assays with *C. difficile*. Interestingly, the MIC (5 mM) of *C. difficile* strains were found to be higher than the MIC of multiple gastrointestinal bacteria tested, ranging from 2–4 mM. These results led the authors to the hypothesis that the ability of *C. difficile* to resist higher gut indole concentrations may provide the bacterium a competitive advantage.^96^ Importantly, these experiments were performed under *in vitro* conditions and future work with animal models is needed to test the proposed hypothesis.

Successful *C. difficile* colonization, occurs when the host gut environment is disrupted. As a result, the bacterium must adapt and take advantage of resources available to maintain colonization and exclude the reestablishment of competing microbes.^97,98^ The ability of the *C. difficile* bacterium to establish a niche in the gut environment by use of metabolites from intestinal epithelial cells, as well as secreted compounds to outcompete other gut microbes, suggests *C. difficile* is a bacterial generalist.^68^ Although the factors that correlate with an environment that excludes *C. difficile* are well documented, the factors that *C. difficile* uses to exclude the microbiome from reestablishing itself are comparatively less understood. By inhibiting these mechanism, the normally-protective microbiome may gain a foothold and return the GI to a colonization resistant environment.

## Conclusions and future directions

This review highlights specific metabolic compounds that have been regarded as important during the life cycle and pathogenesis pathway of CDI. As the research field, moves toward a microbiome-centric study of gut diseases, molecules used as nutritional sources for both pathogen and resident gut bacteria are being identified. In Figure 3, we show graphical representations of microbiome stages that may allow or prevent the gut environment to succumb to a *C. difficile* disease stage, as well as promote recovery.

*C. difficile* pathogenesis is complex. Although much work has focused on the effect that bile acids and antimicrobials have on the bacterium, considering the physiology of *C. difficile* for developing new treatments is emerging in the field. Potential treatment options against CDI have slowly progressed toward approaches that focus on restoring the gut microbiome ecosystem as a whole and not what affects *C. difficile*. Although some progress has been made, there are still many aspects to uncover regarding gut microbiota niche growth and competition in the GI tract. The move from broad-spectrum antibiotic treatment to more specific antibiotics, like ridinilazole that showed fewer CDI recurrences in a phase 2 trial, is happening at present.^99^ Targeted molecules such as nanobodies or DARPins have also emerged and,^100^ will probably become the norm for treatment against CDI in the future. Alternatively, specific strategies to reintroduce defined microbial communities to combat CDI, in the form of targeted microbiome therapies (e.g., SER-109, composed of purified Firmicutes bacterial spores),^101^ might also work as an effective treatment strategy. Unraveling of additional bile salt biotransformations that can be taken advantage of to potentially modulate bile acid composition as well as bile acid analogs that do not undergo enterohepatic recirculation are yet other potential CDI treatments.^45,102^ Finally, introduction of probiotics specifically targeting CDI or synbiotics (probiotics combined with prebiotics) that may modify the gut microbiome by increasing engraftment through introduction of specific nutrient sources appear to have potential for treatment in the future.^103,104^ It is important to mention though, that as understanding of the complex CDI pathogenesis and life cycle expands, the realization that individual treatments options may not be the way forward and comprehensive treatment plans may provide the best results, especially in regards to relapsing *C. difficile* episodes.

## Data Availability

Data sharing is not applicable to this article as no new data were created or analyzed in this study.

## References

[1] Finn E, Andersson FL, Madin-Warburton M. Burden of *Clostridioides difficile* infection (CDI) - a systematic review of the epidemiology of primary and recurrent CDI. BMC Infect Dis. 2021;21:456. doi:10.1186/s12879-021-06147-y. 34016040 PMC8135979

[2] Clancy CJ, Buehrle D, Vu M, Wagener MM, Nguyen MH. Impact of revised infectious diseases Society of America and Society for Healthcare Epidemiology of America clinical practice guidelines on the treatment of *Clostridium difficile* Infections in the United States. Clin Infect Dis. 2021;72:1944–15. doi:10.1093/cid/ciaa484. 32343766

[3] CDC. 2019. Antibiotic resistance threats in the United States, 2019. U.S. Department of Health and Human Services, CDC.

[4] Buffie CG, Bucci V, Stein RR, McKenney PT, Ling L, Gobourne A, No D, Liu H, Kinnebrew M, Viale A, et al. Precision microbiome reconstitution restores bile acid mediated resistance to *Clostridium difficile*. Nature. 2015;517:205–208. doi:10.1038/nature13828. 25337874 PMC4354891

[5] Theriot CM, Koenigsknecht MJ, Carlson PE Jr., Hatton GE, Nelson AM, Li B, Huffnagle GB, Li J, Young VB. Antibiotic-induced shifts in the mouse gut microbiome and metabolome increase susceptibility to *Clostridium difficile* infection. Nat Commun. 2014;5:3114. doi:10.1038/ncomms4114. 24445449 PMC3950275

[6] Shim JO. *Clostridium difficile* in children: to treat or not to treat? Pediatr Gastroenterol Hepatol Nutr. 2014;17:80–84. doi:10.5223/pghn.2014.17.2.80. 25061582 PMC4107224

[7] Bien J, Palagani V, Bozko P. The intestinal microbiota dysbiosis and *Clostridium difficile* infection: is there a relationship with inflammatory bowel disease? Therap Adv Gastroenterol. 2013;6:53–68. doi:10.1177/1756283X12454590. PMC3539291 23320050

[8] Furuya-Kanamori L, Marquess J, Yakob L, Riley TV, Paterson DL, Foster NF, Huber CA, Clements ACA. Asymptomatic Clostridium difficile colonization: epidemiology and clinical implications. BMC Infect Dis. 2015;15:516. doi:10.1186/s12879-015-1258-4. 26573915 PMC4647607

[9] Swick MC, Koehler TM, Driks A. Surviving between hosts: sporulation and transmission. Microbiol Spectr. 2016;4. doi:10.1128/microbiolspec.VMBF-0029-2015. PMC5062746 27726794

[10] Tocheva EI, Ortega DR, Jensen GJ. Sporulation, bacterial cell envelopes and the origin of life. Nat Rev Microbiol. 2016;14:535–542. doi:10.1038/nrmicro.2016.85. 28232669 PMC5327842

[11] Baloh M, Sorg JA. *Clostridioides difficile* spore germination: initiation to DPA release. Curr Opin Microbiol. 2022;65:101–107. doi:10.1016/j.mib.2021.11.001. 34808546 PMC8792321

[12] Deakin LJ, Clare S, Fagan RP, Dawson LF, Pickard DJ, West MR, Wren BW, Fairweather NF, Dougan G, Lawley TD, et al. The Clostridium difficile spo0A gene is a persistence and transmission factor. Infect Immun. 2012;80:2704–2711. doi:10.1128/IAI.00147-12. 22615253 PMC3434595

[13] Jump RL, Pultz MJ, Donskey CJ. Vegetative *Clostridium difficile* survives in room air on moist surfaces and in gastric contents with reduced acidity: a potential mechanism to explain the association between proton pump inhibitors and *C. difficile*-associated diarrhea? Antimicrob Agents Chemother. 2007;51:2883–2887. doi:10.1128/AAC.01443-06. 17562803 PMC1932506

[14] Barra-Carrasco J, Hernandez-Rocha C, Ibanez P, Guzman-Duran AM, Alvarez-Lobos M, Paredes-Sabja D. *Clostridium difficile* spores and its relevance in the persistence and transmission of the infection. Rev Chilena Infectol. 2014;31:694–703. doi:10.4067/S0716-10182014000600010. 25679927

[15] Bhattacharjee D, McAllister KN, Sorg JA. Germinants and their receptors in Clostridia. J Bacteriol. 2016;198:2767–2775. doi:10.1128/JB.00405-16. 27432831 PMC5038010

[16] Sorg JA, Sonenshein AL. Bile salts and glycine as cogerminants for *Clostridium difficile* spores. J Bacteriol. 2008;190:2505–2512. doi:10.1128/JB.01765-07. 18245298 PMC2293200

[17] Orrell KE, Zhang Z, Sugiman-Marangos SN, Melnyk RA. *Clostridium difficile* toxins A and B: receptors, pores, and translocation into cells. Crit Rev Biochem Mol Biol. 2017;52:461–473. doi:10.1080/10409238.2017.1325831. 28545305

[18] Aktories K, Schwan C, Jank T. *Clostridium difficile* Toxin Biology. Annu Rev Microbiol. 2017;71:281–307. doi:10.1146/annurev-micro-090816-093458. 28657883

[19] Di Bella S, Ascenzi P, Siarakas S, Petrosillo N, and Di Masi A. *Clostridium difficile* toxins A and B: insights into pathogenic properties and extraintestinal effects. Toxins (Basel). 2016;8(5);134. doi:10.3390/toxins8050134. PMC4885049 27153087

[20] Perelle S, Gibert M, Bourlioux P, Corthier G, Popoff MR. Production of a complete binary toxin (actin-specific ADP- ribosyltransferase) by *Clostridium difficile* CD196. Infect Immun. 1997;65:1402–1407. doi:10.1128/iai.65.4.1402-1407.1997. 9119480 PMC175146

[21] Eyre DW, Walker AS, Griffiths D, Wilcox MH, Wyllie DH, Dingle KE, Crook DW, Peto TE. *Clostridium difficile* mixed infection and reinfection. J Clin Microbiol. 2012;50:142–144. doi:10.1128/JCM.05177-11. 22075589 PMC3256686

[22] Shoff CJ, Spires SS, Yen C, Advani SD. Navigating the 2021 update to the IDSA/SHEA *Clostridioides difficile* guidelines: an ethical approach to equitable patient care. Antimicrob Stewardship Healthcare Epidemiol. 2022;2:e70. doi:10.1017/ash.2022.49. PMC9726573 36483335

[23] Xiao Y, Angulo MT, Lao S, Weiss ST, Liu YY. An ecological framework to understand the efficacy of fecal microbiota transplantation. Nat Commun. 2020;11:3329. doi:10.1038/s41467-020-17180-x. 32620839 PMC7334230

[24] DeFilipp Z, Bloom PP, Torres Soto M, Mansour MK, Sater MRA, Huntley MH, Turbett S, Chung RT, Chen YB, Hohmann EL, et al. Drug-resistant *E. coli* bacteremia transmitted by fecal microbiota transplant. N Engl J Med. 2019;381:2043–2050. doi:10.1056/NEJMoa1910437. 31665575

[25] Khoruts A. Is fecal microbiota transplantation a temporary patch for treatment of *Clostridium difficile* infection or a new frontier of therapeutics? Expert Rev Gastroenterol Hepatol. 2018;12:435–438. doi:10.1080/17474124.2018.1465818. 29667436

[26] Jiang ZD, Ajami NJ, Petrosino JF, Jun G, Hanis CL, Shah M, Hochman L, Ankoma-Sey V, DuPont AW, Wong MC, et al. Randomised clinical trial: faecal microbiota transplantation for recurrent *Clostridum difficile* infection - fresh, or frozen, or lyophilised microbiota from a small pool of healthy donors delivered by colonoscopy. Aliment Pharmacol Ther. 2017;45:899–908. doi:10.1111/apt.13969. 28220514

[27] Chiang JY. Regulation of bile acid synthesis: pathways, nuclear receptors, and mechanisms. J Hepatol. 2004;40:539–551. doi:10.1016/j.jhep.2003.11.006. 15123373

[28] Molinaro A, Wahlstrom A, Marschall HU. Role of bile acids in metabolic control. Trends Endocrinol Metab. 2018;29:31–41. doi:10.1016/j.tem.2017.11.002. 29195686

[29] John YL, and Chiang JMF. Bile acid biology, pathophysiology, and therapeutics. Clin Liver Dis. 2020;15(3):91–94. doi:10.1002/cld.861. PMC7128030 32257118

[30] Ridlon JM, Kang DJ, Hylemon PB, Bajaj JS. Bile acids and the gut microbiome. Curr Opin Gastroenterol. 2014;30:332–338. doi:10.1097/MOG.0000000000000057. 24625896 PMC4215539

[31] Ridlon JM, Harris SC, Bhowmik S, Kang DJ, Hylemon PB. Consequences of bile salt biotransformations by intestinal bacteria. Gut Microbes. 2016;7:22–39. doi:10.1080/19490976.2015.1127483. 26939849 PMC4856454

[32] Kliewer SA, Mangelsdorf DJ. Bile acids as hormones: the FXR-FGF15/19 pathway. Dig Dis. 2015;33:327–331. doi:10.1159/000371670. 26045265 PMC4465534

[33] Reue K, Lee JM, Vergnes L. Regulation of bile acid homeostasis by the intestinal Diet1-FGF15/19 axis. Curr Opin Lipidol. 2014;25:140–147. doi:10.1097/MOL.0000000000000060. 24535283 PMC4497822

[34] Monaghan T, Mullish BH, Patterson J, Wong GKS, Marchesi JR, Xu H, Jilani T, Kao D. Effective fecal microbiota transplantation for recurrent *Clostridioides difficile* infection in humans is associated with increased signalling in the bile acid-farnesoid X receptor-fibroblast growth factor pathway. Gut Microbes. 2019;10:142–148. doi:10.1080/19490976.2018.1506667. 30183484 PMC6546339

[35] Jose S, Mukherjee A, Horrigan O, Setchell KDR, Zhang W, Moreno-Fernandez ME, Andersen H, Sharma D, Haslam DB, Divanovic S, et al. Obeticholic acid ameliorates severity of *Clostridioides difficile* infection in high fat diet-induced obese mice. Mucosal Immunol. 2021;14:500–510. doi:10.1038/s41385-020-00338-7. 32811993 PMC7889747

[36] Winston JA, Rivera AJ, Cai J, Thanissery R, Montgomery SA, Patterson AD, Theriot, CW. Ursodeoxycholic acid (UDCA) mitigates the host inflammatory response during *Clostridioides difficile* infection by altering gut bile acids. Infect Immun. 2020;88:e00045–20. doi:10.1128/IAI.00045-20. 32205405 PMC7240095

[37] Wilson KH, Kennedy MJ, Fekety FR. Use of sodium taurocholate to enhance spore recovery on a medium selective for *Clostridium difficile*. J Clin Microbiol. 1982;15:443–446. doi:10.1128/jcm.15.3.443-446.1982. 7076817 PMC272115

[38] Sorg JA, Sonenshein AL. Chenodeoxycholate is an inhibitor of *Clostridium difficile* spore germination. J Bacteriol. 2009;191:1115–1117. doi:10.1128/JB.01260-08. 19060152 PMC2632082

[39] Francis MB, Allen CA, Sorg JA. Muricholic acids inhibit *Clostridium difficile* spore germination and growth. PLoS One. 2013;8:e73653. doi:10.1371/journal.pone.0073653. 24040011 PMC3767737

[40] Weingarden AR, Chen C, Zhang N, Graiziger CT, Dosa PI, Steer CJ, Shaughnessy MK, Johnson JR, Sadowsky MJ, Khoruts A, et al. Ursodeoxycholic acid inhibits *Clostridium difficile* spore germination and vegetative growth, and prevents the recurrence of ileal pouchitis associated with the infection. J Clin Gastroenterol. 2016;50:624–630. doi:10.1097/MCG.0000000000000427. 26485102 PMC4834285

[41] Weingarden AR, Chen C, Bobr A, Yao D, Lu Y, Nelson VM, Sadowsky MJ, Khoruts A. Microbiota transplantation restores normal fecal bile acid composition in recurrent *Clostridium difficile* infection. Am J Physiol Gastrointest Liver Physiol. 2014;306:G310–9. doi:10.1152/ajpgi.00282.2013. 24284963 PMC3920123

[42] Chen X, Katchar K, Goldsmith JD, Nanthakumar N, Cheknis A, Gerding DN, Kelly CP. A mouse model of *Clostridium difficile*–associated disease. Gastroenterology. 2008;135:1984–1992. doi:10.1053/j.gastro.2008.09.002. 18848941

[43] Hofmann AF. The continuing importance of bile acids in liver and intestinal disease. Arch Intern Med. 1999;159:2647–2658. doi:10.1001/archinte.159.22.2647. 10597755

[44] Thanissery R, Winston JA, Theriot CM. Inhibition of spore germination, growth, and toxin activity of clinically relevant *C. difficile* strains by gut microbiota derived secondary bile acids. Anaerobe. 2017;45:86–100. doi:10.1016/j.anaerobe.2017.03.004. 28279860 PMC5466893

[45] Sato Y, Atarashi K, Plichta DR, Arai Y, Sasajima S, Kearney SM, Suda W, Takeshita K, Sasaki T, Okamoto S, et al. Novel bile acid biosynthetic pathways are enriched in the microbiome of centenarians. Nature. 2021;599:458–464. doi:10.1038/s41586-021-03832-5. 34325466

[46] Wilson KH. Efficiency of various bile salt preparations for stimulation of *Clostridium difficile* spore germination. J Clin Microbiol. 1983;18:1017–1019. doi:10.1128/jcm.18.4.1017-1019.1983. 6630458 PMC270959

[47] Winston JA, Theriot CM. Impact of microbial derived secondary bile acids on colonization resistance against *Clostridium difficile* in the gastrointestinal tract. Anaerobe. 2016;41:44–50. doi:10.1016/j.anaerobe.2016.05.003. 27163871 PMC5050083

[48] Aguirre AM, Yalcinkaya N, Wu Q, Swennes A, Tessier ME, Roberts P, Miyajima F, Savidge T, and Sorg JA. Bile acid-independent protection against *Clostridioides difficile* infection. PLoS Pathog. 2021;17:e1010015. doi:10.1371/journal.ppat.1010015. 34665847 PMC8555850

[49] Seekatz AM, Theriot CM, Rao K, Chang YM, Freeman AE, Kao JY, Young VB. Restoration of short chain fatty acid and bile acid metabolism following fecal microbiota transplantation in patients with recurrent *Clostridium difficile* infection. Anaerobe. 2018;53:64–73. doi:10.1016/j.anaerobe.2018.04.001. 29654837 PMC6185828

[50] Staley C, Vaughn BP, Graiziger CT, Singroy S, Hamilton MJ, Yao D, Chen C, Khoruts A, Sadowsky MJ. Community dynamics drive punctuated engraftment of the fecal microbiome following transplantation using freeze-dried, encapsulated fecal microbiota. Gut Microbes. 2017;8:276–288. doi:10.1080/19490976.2017.1299310. 28282270 PMC5479395

[51] Wexler AG, Guiberson ER, Beavers WN, Shupe JA, Washington MK, Lacy DB, Caprioli RM, Spraggins JM, Skaar EP. *Clostridioides difficile* infection induces a rapid influx of bile acids into the gut during colonization of the host. Cell Rep. 2021;36:109683. doi:10.1016/j.celrep.2021.109683. 34496241 PMC8445666

[52] Dubois T, Tremblay YDN, Hamiot A, Martin-Verstraete I, Deschamps J, Monot M, Briandet R, Dupuy B. A microbiota-generated bile salt induces biofilm formation in *Clostridium difficile*. NPJ Biofilms Microbiomes. 2019;5:14. doi:10.1038/s41522-019-0087-4. 31098293 PMC6509328

[53] Sievers S, Metzendorf NG, Dittmann S, Troitzsch D, Gast V, Troger SM, Wolff C, Zuhlke D, Hirschfeld C, Schulter R, Riedel K, et al. Differential view on the bile acid stress response of *Clostridioides difficile*. Front Microbiol. 2019;10:258. doi:10.3389/fmicb.2019.00258. 30833939 PMC6387971

[54] Tam J, Icho S, Utama E, Orrell KE, Gómez-Biagi RF, Theriot CM, Kroh HK, Rutherford SA, Lacy DB, Melnyk RA, et al. Intestinal bile acids directly modulate the structure and function of *C. difficile* TcdB toxin. Proc Natl Acad Sci. 2020;117:6792–6800. doi:10.1073/pnas.1916965117. 32152097 PMC7104382

[55] Stickland LH. Studies in the metabolism of the strict anaerobes (genus Clostridium). Biochem J. 1934;28:1746–1759. doi:10.1042/bj0281746. 16745572 PMC1253397

[56] Nisman BRM, Cohen GN, COHEN GN. Extension of the Stickland reaction to several bacterial species. Arch Biochem. 1948;16:473. 18903718

[57] LH S. Studies in the metabolism of the strict anaerobes (genus Clostridium)- The oxidation of alanine by Cl. sporogenes. IV. The reduction of glycine by Cl. sporogenes. Biochem J. 1935;29:889–898. doi:10.1042/bj0290889. 16745741 PMC1266567

[58] Jackson S, Calos M, Myers A, Self WT. Analysis of proline reduction in the nosocomial pathogen *Clostridium difficile*. J Bacteriol. 2006;188(24):8487–8495. doi:10.1128/JB.01370-06. 17041035 PMC1698225

[59] Bouillaut L, Self WT, Sonenshein AL. Proline-dependent regulation of *Clostridium difficile* Stickland metabolism. J Bacteriol. 2013;195:844–854. doi:10.1128/JB.01492-12. 23222730 PMC3562115

[60] Fonknechten Nuria CS, Sabine T, Aurélie L, Andreesen Jan R, Nadia P, Eric P, Michel G, Valérie B, Marcel S, Le Paslier D, et al. *Clostridium sticklandii*, a specialist in amino acid degradation:revisiting its metabolism through its genome sequence. BMC Genomics. 2010;11. doi:10.1186/1471-2164-11-555. PMC3091704 20937090

[61] Bouillaut L, Dubois T, Francis MB, Daou N, Monot M, Sorg JA, Sonenshein AL, Dupuy B. Role of the global regulator Rex in control of NAD + -regeneration in Clostridioides (Clostridium) difficile. Mol Microbiol. 2019;111:1671–1688. doi:10.1111/mmi.14245. 30882947 PMC6561804

[62] Self WT. Specific and nonspecific incorporation of selenium into macromolecules. Comprehensive Natural Products Ii: Chemistry and Biology, Vol 5: Amino Acids, Peptides and Proteins. 2010;121–148.

[63] McAllister KN, Bouillaut L, Kahn JN, Self WT, Sorg JA. Using CRISPR-Cas9-mediated genome editing to generate *C. difficile* mutants defective in selenoproteins synthesis. Sci Rep. 2017;7:14672. doi:10.1038/s41598-017-15236-5. 29116155 PMC5677094

[64] McAllister KN, Martinez Aguirre A, Sorg JA. The Selenophosphate Synthetase Gene, *selD*, Is Important for *Clostridioides difficile* physiology. J Bacteriol. 2021;203:e0000821. doi:10.1128/JB.00008-21. 33820795 PMC8315937

[65] Battaglioli EJ, Hale VL, Chen J, Jeraldo P, Ruiz-Mojica C, Schmidt BA, Rekdal VM, Till LM, Huq L, Smits SA, Moor WJ, Hall YJ, Smyrk T, Khanna S, Pardi DS, Grover M, Patel R, Chia N, Nelson H, Sonnenburg JL, and Farrugia G, et al. *Clostridioides difficile* uses amino acids associated with gut microbial dysbiosis in a subset of patients with diarrhea. Sci Transl Med. 2018;10. doi:10.1126/scitranslmed.aam7019 PMC6537101 30355801

[66] Girinathan BP, DiBenedetto N, Worley JN, Peltier J, Arrieta-Ortiz ML, Immanuel SRC, Lavin R, Delaney ML, Cummins CK, Hoffman M, et al. In vivo commensal control of *Clostridioides difficile* virulence. Cell Host Microbe. 2021;29:1693–708 e7. doi:10.1016/j.chom.2021.09.007. 34637781 PMC8651146

[67] Robinson JI, Weir WH, Crowley JR, Hink T, Reske KA, Kwon JH, Burnham CAD, Dubberke ER, Mucha PJ, Henderson JP, et al. Metabolomic networks connect host-microbiome processes to human *Clostridioides difficile* infections. J Clin Invest. 2019;129:3792–3806. 31403473 10.1172/JCI126905 PMC6715368

[68] Jenior ML, JL L, Young BV, Schloss PD. *Clostridium difficile* colonizes alternative nutrient niches during infection across distinct murine gut microbiomes. mSystems. 2017;2. doi:10.1128/mSystems.00063-17. PMC5527303 28761936

[69] Hofmann JD, Biedendieck R, Michel AM, Schomburg D, Jahn D, Neumann-Schaal M. Influence of L-lactate and low glucose concentrations on the metabolism and the toxin formation of *Clostridioides difficile*. PLoS One. 2021;16:e0244988. doi:10.1371/journal.pone.0244988. 33411772 PMC7790285

[70] Fletcher JR, Pike CM, Parsons RJ, Rivera AJ, Foley MH, McLaren MR, Montgomery SA, Theriot CM, et al. *Clostridioides difficile* exploits toxin-mediated inflammation to alter the host nutritional landscape and exclude competitors from the gut microbiota. Nat Commun. 2021;12:462. doi:10.1038/s41467-020-20746-4. 33469019 PMC7815924

[71] Hromada S, Qian Y, Jacobson TB, Clark RL, Watson L, Safdar N, Amador-Noguez D, Venturelli OS, et al. Negative interactions determine *Clostridioides difficile* growth in synthetic human gut communities. Mol Syst Biol. 2021;17:e10355. doi:10.15252/msb.202110355. 34693621 PMC8543057

[72] Bajaj JS, Kakiyama G, Savidge T, Takei H, Kassam ZA, Fagan A, Gavis EA, Pandak WM, Nittono H, Hylemon PB, Boonma P, Haag A, Heuman DM, Fuchs M, John B, Sikaroodi M, Gillevet PM, et al. Antibiotic-associated disruption of microbiota composition and function in cirrhosis is restored by fecal transplant. Hepatology. 2018;68:1549–1558. doi:10.1002/hep.30037. 29665102

[73] Cheng S, M X, Geng S, Jiang X, Yuan L, Luansha H, Jianrong L, Wang Y, and Han X. Fecal microbiota transplantation beneficially regulates intestinal mucosal autophagy and alleviates gut barrier injury. mSystems. 2018;3(5):e00137–18. doi:10.1128/mSystems.00137-18. 30320222 PMC6178585

[74] Littmann ER, Lee JJ, Denny JE, Alam Z, Maslanka JR, Zarin I, Matsuda R, Carter RA, Susac B, Saffern MS, et al. Host immunity modulates the efficacy of microbiota transplantation for treatment of *Clostridioides difficile* infection. Nat Commun. 2021;12:755. doi:10.1038/s41467-020-20793-x. 33531483 PMC7854624

[75] McDonald JAK, Mullish BH, Pechlivanis A, Liu Z, Brignardello J, Kao D, Holmes E, Li JV, Clarke TB, Thursz MR, et al. Inhibiting growth of *Clostridioides difficile* by restoring valerate, produced by the intestinal microbiota. Gastroenterology. 2018;155:1495–507 e15. doi:10.1053/j.gastro.2018.07.014. 30025704 PMC6347096

[76] Neumann-Schaal M, Jahn D, Schmidt-Hohagen K. Metabolism the difficile way: the key to the success of the pathogen *Clostridioides difficile*. Front Microbiol. 2019;10:219. doi:10.3389/fmicb.2019.00219. 30828322 PMC6384274

[77] Gencic S, and Grahame DA. Diverse energy-conserving pathways in *Clostridium difficile*: growth in the absence of amino acid Stickland acceptors and the role of the wood-Ljungdahl pathway. J Bacteriol. 2020:202(20):e00233–20. doi:10.1128/JB.00233-20. 32967909 PMC7515248

[78] Koenigsknecht MJ, Theriot CM, Bergin IL, Schumacher CA, Schloss PD, Young VB. Dynamics and establishment of *Clostridium difficile* infection in the murine gastrointestinal tract. Infect Immun. 2015;83:934–941. doi:10.1128/IAI.02768-14. 25534943 PMC4333439

[79] Lopez CA, McNeely TP, Nurmakova K, Beavers WN, Skaar EP. *Clostridioides difficile* proline fermentation in response to commensal clostridia. Anaerobe. 2020;63:102210. doi:10.1016/j.anaerobe.2020.102210. 32422411 PMC8025294

[80] Kriaa A, Bourgin M, Mkaouar H, Jablaoui A, Akermi N, Soussou S, Maguin E, Rhimi M. Microbial reduction of cholesterol to Coprostanol: an old concept and new insights. Catalysts. 2019;9. doi:10.3390/catal9020167.

[81] Antharam VC, McEwen DC, Garrett TJ, Dossey AT, Li EC, Kozlov AN, Mesbah Z, Wang GP. An integrated metabolomic and microbiome analysis identified specific gut microbiota associated with fecal cholesterol and coprostanol in *Clostridium difficile* infection. PLoS One. 2016;11:e0148824. doi:10.1371/journal.pone.0148824. 26871580 PMC4752508

[82] Jump RL, Polinkovsky A, Hurless K, Sitzlar B, Eckart K, Tomas M, Deshpande A, Nerandzic MM, Donskey CJ. Metabolomics analysis identifies intestinal microbiota-derived biomarkers of colonization resistance in clindamycin-treated mice. PLoS One. 2014;9:e101267. doi:10.1371/journal.pone.0101267. 24988418 PMC4079339

[83] Kellingray L, Gall GL, Defernez M, Beales ILP, Franslem-Elumogo N, Narbad A. Microbial taxonomic and metabolic alterations during faecal microbiota transplantation to treat *Clostridium difficile* infection. J Infect. 2018;77:107–118. doi:10.1016/j.jinf.2018.04.012. 29746938

[84] Lavelle A, Sokol H. Gut microbiota-derived metabolites as key actors in inflammatory bowel disease. Nat Rev Gastroenterol Hepatol. 2020;17:223–237. doi:10.1038/s41575-019-0258-z. 32076145

[85] Martinez-Gili L, McDonald JAK, Liu Z, Kao D, Allegretti JR, Monaghan TM, Barker GF, Blanco JM, Williams HRT, Holmes E, Thursz MR, Marchesi JR, Mullish BH, et al. Understanding the mechanisms of efficacy of fecal microbiota transplant in treating recurrent *Clostridioides difficile* infection and beyond: the contribution of gut microbial-derived metabolites. Gut Microbes. 2020;12:1810531. doi:10.1080/19490976.2020.1810531. 32893721 PMC7524310

[86] Garland M, Hryckowian AJ, Tholen M, Bender KO, Van Treuren WW, Loscher S, Sonnenburg JL, Bogyo M. The clinical drug ebselen attenuates inflammation and promotes microbiome recovery in mice after antibiotic treatment for CDI. Cell Rep Med. 2020;1(1):100005. doi:10.1016/j.xcrm.2020.100005. 32483557 PMC7263476

[87] Fachi JL, Felipe JS, Pral LP, da Silva BK, Correa RO, de Andrade MCP, da Fonseca DM, Basso PJ, Câmara NOS, de Sales E Souza ÉL, et al. Butyrate protects mice from *Clostridium difficile*-induced colitis through an HIF-1-dependent mechanism. Cell Rep. 2019;27:750–61 e7. doi:10.1016/j.celrep.2019.03.054. 30995474

[88] Fachi JL, Secca C, Rodrigues PB, Mato FCP, Di Luccia B, Felipe JS, Pral LP, Rungue M, Rocha VDM, Sato FT, et al. Acetate coordinates neutrophil and ILC3 responses against *C. difficile* through FFAR2. J Exp Med. 2020;217. doi:10.1084/jem.20190489 PMC7062529 31876919

[89] Gregory AL, Pensinger DA, Hryckowian AJ. A short chain fatty acid-centric view of *Clostridioides difficile* pathogenesis. PLoS Pathog. 2021;17:e1009959. doi:10.1371/journal.ppat.1009959. 34673840 PMC8530303

[90] Kang JD, Myers CJ, Harris SC, Kakiyama G, Lee IK, Yun BS, Matsuzaki K, Furukawa M, Min HK, Bajaj JS, Zhou H, and Hylemon PB, et al. Bile acid 7alpha-dehydroxylating gut bacteria secrete antibiotics that inhibit *Clostridium difficile*: role of secondary bile acids. Cell Chem Biol. 2019;26:27–34 e4. doi:10.1016/j.chembiol.2018.10.003. 30482679 PMC6338514

[91] Saa P, Urrutia A, Silva-Andrade C, Martín AJ, Garrido D. Modeling approaches for probing cross-feeding interactions in the human gut microbiome. Comput Struct Biotechnol J. 2022;20:79–89. doi:10.1016/j.csbj.2021.12.006. 34976313 PMC8685919

[92] Knippel RJ, Wexler AG, Miller JM, Beavers WN, Weiss A, de Crecy-Lagard V, Edmonds KA, Giedroc DP, Skaar EP. *Clostridioides difficile* senses and hijacks host heme for incorporation into an oxidative stress defense system. Cell Host Microbe. 2020;28:411–21 e6. doi:10.1016/j.chom.2020.05.015. 32526159 PMC7486240

[93] Passmore IJ, Letertre MPM, Preston MD, Bianconi I, Harrison MA, Nasher F, Kaur H, Hong HA, Baines SD, Cutting SM, Swann JR, Wren BW, Dawson LF. Para-cresol production by *Clostridium difficile* affects microbial diversity and membrane integrity of gram-negative bacteria. PLoS Pathog. 2018;14:e1007191. doi:10.1371/journal.ppat.1007191. 30208103 PMC6135563

[94] Pruss KM, Sonnenburg JL. *C. difficile* exploits a host metabolite produced during toxin-mediated disease. Nature. 2021;593:261–265. doi:10.1038/s41586-021-03502-6. 33911281 PMC9067157

[95] Ng KM, Ferreyra JA, Higginbottom SK, Lynch JB, Kashyap PC, Gopinath S, Naidu N, Choudhury B, Weimer BC, Monack DM, Sonnenburg JL. Microbiota-liberated host sugars facilitate post-antibiotic expansion of enteric pathogens. Nature. 2013;502:96–99. doi:10.1038/nature12503. 23995682 PMC3825626

[96] Darkoh C, Plants-Paris K, Bishoff D, and DuPont HL. *Clostridium difficile* modulates the gut microbiota by inducing the production of indole, an interkingdom signaling and antimicrobial molecule. mSystems. 2019;4(2):e00346–18. 30944877 10.1128/mSystems.00346-18 PMC6426650

[97] Donskey CJ, Kundrapu S, Deshpande A. Colonization versus carriage of *Clostridium difficile*. Infect Dis Clin North Am. 2015;29:13–28. doi:10.1016/j.idc.2014.11.001. 25595843

[98] Janoir C, Deneve C, Bouttier S, Barbut F, Hoys S, Caleechum L, Chapeton-Montes D, Pereira FC, Henriques AO, Collignon A, Monot M, Dupuy B, et al. Adaptive strategies and pathogenesis of *Clostridium difficile* from in vivo transcriptomics. Infect Immun. 2013;81:3757–3769. doi:10.1128/IAI.00515-13. 23897605 PMC3811758

[99] Qian X, Yanagi K, Kane AV, Alden N, Lei M, Snydman DR, Vickers RJ, Lee K, Thorpe CM. Ridinilazole, a narrow spectrum antibiotic for treatment of *Clostridioides difficile infection*, enhances preservation of microbiota-dependent bile acids. Am J Physiol Gastrointest Liver Physiol. 2020;319:G227–G37. doi:10.1152/ajpgi.00046.2020. 32597706 PMC7500266

[100] Simeon RA, Zeng Y, Chonira V, Aguirre AM, Lasagna M, Baloh M, Sorg JA, Tommos C, Chen Z. Protease-stable DARPins as promising oral therapeutics. Protein Eng Des Sel. 2021;34. doi:10.1093/protein/gzab028. PMC8861517 34882774

[101] Feuerstadt P, Louie TJ, Lashner B, Wang EEL, Diao L, Bryant JA, Sims M, Kraft CS, Cohen SH, Berenson CS, et al. SER-109, an oral microbiome therapy for recurrent *Clostridioides difficile* Infection. N Engl J Med. 2022;386:220–229. doi:10.1056/NEJMoa2106516. 35045228

[102] Stoltz KL, Erickson R, Staley C, Weingarden AR, Romens E, Steer CJ, Khoruts A, Sadowsky MJ, Dosa PI. Synthesis and biological evaluation of bile acid analogues inhibitory to *Clostridium difficile* spore germination. J Med Chem. 2017;60:3451–3471. doi:10.1021/acs.jmedchem.7b00295. 28402634 PMC5877410

[103] Hryckowian AJ, Van Treuren W, Smits SA, Davis NM, Gardner JO, Bouley DM, Sonnenburg JL. Microbiota-accessible carbohydrates suppress *Clostridium difficile* infection in a murine model. Nat Microbiol. 2018;3:662–669. doi:10.1038/s41564-018-0150-6. 29686297 PMC6126909

[104] Rätsep M, Kõljalg S, Sepp E, Smidt I, Truusalu K, Songisepp E, Stsepetova J, Naaber P, Mikelsaar RH, Mikelsaar M, et al. A combination of the probiotic and prebiotic product can prevent the germination of *Clostridium difficile* spores and infection. Anaerobe. 2017;47:94–103. doi:10.1016/j.anaerobe.2017.03.019. 28465256

